# Lysine Auxotrophy Combined with Deletion of the *SecA2* Gene Results in a Safe and Highly Immunogenic Candidate Live Attenuated Vaccine for Tuberculosis

**DOI:** 10.1371/journal.pone.0015857

**Published:** 2011-01-10

**Authors:** Joseph Hinchey, Bo Y. Jeon, Holly Alley, Bing Chen, Michael Goldberg, Steven Derrick, Sheldon Morris, William R. Jacobs, Steven A. Porcelli, Sunhee Lee

**Affiliations:** 1 Department of Microbiology and Immunology, Albert Einstein College of Medicine, Bronx, New York, United States of America; 2 Department of Microbiology, Yonsei University College of Medicine, Seoul, Republic of Korea; 3 Duke Human Vaccine Institute, Durham, North Carolina, United States of America; 4 Center for Biologics Evaluation, US Food and Drug Administration, Bethesda, Maryland, United States of America; 5 Howard Hughes Medical Institute, Albert Einstein College of Medicine, Bronx, New York, United States of America; 6 Department of Medicine, Albert Einstein College of Medicine, Bronx, New York, United States of America; University of Sao Paulo, Brazil

## Abstract

Tuberculosis (TB) caused by *Mycobacterium tuberculosis* remains a major global health problem, despite the widespread use of the *M. bovis* Bacille Calmette-Guerin (BCG) vaccine and the availability of drug therapies. In recent years, the high incidence of coinfection of *M. tuberculosis* and HIV, as well as escalating problems associated with drug resistance, has raised ominous concerns with regard to TB control. Vaccination with BCG has not proven highly effective in controlling TB, and also has been associated with increasing concerns about the potential for the vaccine to cause disseminated mycobacterial infection in HIV infected hosts. Thus, the development of an efficacious and safe TB vaccine is generally viewed as a critical to achieving control of the ongoing global TB pandemic. In the current study, we have analyzed the vaccine efficacy of an attenuated *M. tuberculosis* strain that combines a mutation that enhances T cell priming (Δ*secA2*) with a strongly attenuating lysine auxotrophy mutation (Δ*lysA*). The Δ*secA2* mutant was previously shown to be defective in the inhibition of apoptosis and markedly increased priming of antigen-specific CD8^+^ T cells *in vivo*. Similarly, the Δ*secA2*Δ*lysA* strain retained enhanced apoptosis and augmented CD8^+^ T cell stimulatory effects, but with a noticeably improved safety profile in immunosuppressed mice. Thus, the *M. tuberculosis* Δ*secA2*Δ*lysA* mutant represents a live attenuated TB vaccine strain with the potential to deliver increased protection and safety compared to standard BCG vaccination.

## Introduction

Tuberculosis is a devastating disease, and despite the availability of therapeutic drugs and widespread vaccination with BCG, it remains a serious global health issue, infecting nearly a third of the world population and causing 2–3 million deaths annually [1]. Since initiating BCG vaccination in the 1920s, more than three billion people have been vaccinated with BCG [2]. However, the benefits and drawbacks of BCG have been debated since its early use. Ongoing concerns and controversies include the potential for disseminated infection by BCG in immunosuppressed hosts, loss of sensitivity to tuberculin as a diagnostic reagent, and the failure of BCG to show efficacy in the prevention of pulmonary TB in a number of trials in developing countries [3], [4], [5]. There have been numerous hypotheses to explain the inadequate protective effect of BCG against pulmonary TB. Factors related directly to the vaccine, such as inappropriate treatment and storage of the vaccine and strain variability have been suggested. Additionally, several deficits of a more fundamental immunological character have been proposed, such as a lack of important antigens, interaction with environmental mycobacteria, and dwindling efficacy of BCG vaccination over time [6], [7]. Because various T cell subsets have been shown to participate in the immune responses against mycobacteria, the lack of an effective stimulation of the optimal blend of T cell populations, and particularly the failure to induce CD8^+^ T cells, may also explain the insufficient levels of immunity promoted by the BCG vaccine strains [7], [8]. In fact, increased vaccine efficacy against tuberculosis has been observed in association with enhanced priming of CD8^+^ T cells *in vivo* [9], [10]. As there is an urgent need for innovative protective vaccine strategies, we must identify and exploit the mechanisms that can facilitate or inhibit immunity to *M. tuberculosis*, while simultaneously maintaining an adequate safety profile.

In previous work, we described a novel immune-evasion strategy of *M. tuberculosis* by which secretion of superoxide dismutase A (SodA) inhibits the apoptotic program of infected macrophages, effectively blunting the specific CD8^+^ T-cell responses to the bacterium. In those studies, inactivation of the *M. tuberculosis secA2* gene, which encodes a virulence-associated protein secretion system, enhanced the apoptosis of infected macrophages and increased priming of antigen-specific CD8^+^ T cells *in vivo*. Vaccination of mice and guinea pigs with the Δ*secA2* mutant significantly increased resistance to virulent *M. tuberculosis* challenge [9]. Thus, inactivation of the *secA2* gene represents a novel way to improve the immunogenicity of mycobacterial vaccines. However, in subsequent studies we observed that inactivation of *secA2* in BCG did not lead to enhanced immunogenicity (unpublished data) and the Δ*secA2* mutant retained a moderate level of virulence, making it unsuitable as a vaccine. In order to improve vaccine efficacy and safety of the Δ*secA2* mutant, we generated multiple double and triple mutants of the Δ*secA2* mutant strain via incorporation of other attenuating mutations of *M. tuberculosis*. In the current study, we have evaluated one of the double mutants, Δ*secA2*Δ*lysA*, and show here that this strain displays an excellent safety profile in immunocompromised SCID mice while retaining the enhanced immunogenicity of the parental Δ*secA2* mutant strain.

## Results

### Improved attenuation of *M. tuberculosis* Δ*secA2*Δ*lysA* in SCID mice

**Figure 1:**
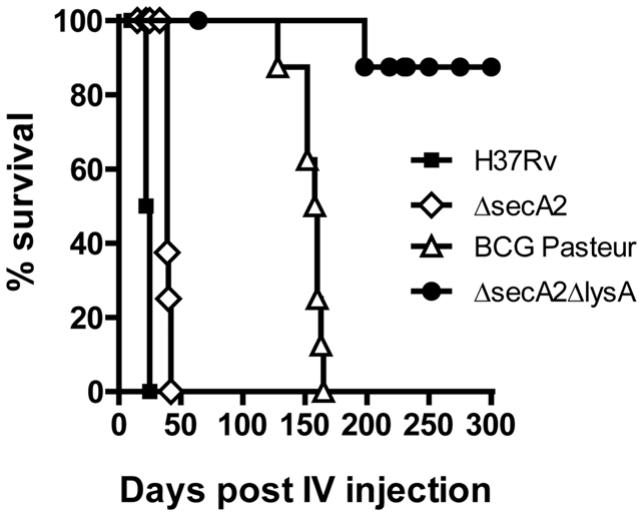
Survival of immunocompromised mice after infection with virulent *M. tuberculosis*, *ΔsecA2, ΔsecA2ΔlysA*, or BCG. SCID mice were infected intravenously with 10^5^ CFUs of virulent *M. tuberculosis* (H37Rv), *M. tuberculosis ΔsecA2,* BCG Pasteur, or *M. tuberculosis ΔsecA2ΔlysA*. Mice were followed for survival, or were sacrificed humanely when moribund.

The principal advantage of incorporating auxotrophic mutations into vaccine candidates is the associated reduction in virulence. Previously, the *ΔlysA* strain of *M. tuberculosis* was shown to be unable to replicate *in vitro* in the absence of lysine supplementation of the growth medium, and to be markedly attenuated for growth *in vivo* in SCID mice, consistent with the inability of the bacteria to scavenge sufficient lysine from the intracellular environment [11], [12]. Strain Δ*secA2*Δ*lysA* is strictly auxotrophic for lysine, and no growth of the mutant was observed in the absence of lysine supplementation. To investigate the virulence of the double mutant, SCID mice were injected intravenously with 10^5^ CFU of either wild-type *M. tuberculosis* (strain H37Rv), a Δ*secA2* mutant, or a double mutant strain *ΔsecA2ΔlysA.* As expected, the Δ*secA2* single mutant of *M. tuberculosis* showed a high level of virulence in this experiment, as all animals died in less than 50 days. In contrast, a significant reduction of virulence of the Δ*secA2*Δ*lysA* strain was observed, as the group infected with this strain had a median survival of >300 days. In comparison, BCG infected SCID mice showed a median survival of ∼150 days, and all BCG infected animals succumbed by day 165 (Figure 1). These data clearly indicated that *ΔsecA2ΔlysA* was strongly attenuated and even less virulent than the standard BCG vaccine strain in the context of severe immunodeficiency.

### Induction of macrophage apoptosis by *M. tuberculosis* Δ*secA2*Δ*lysA*

**Figure 2:**
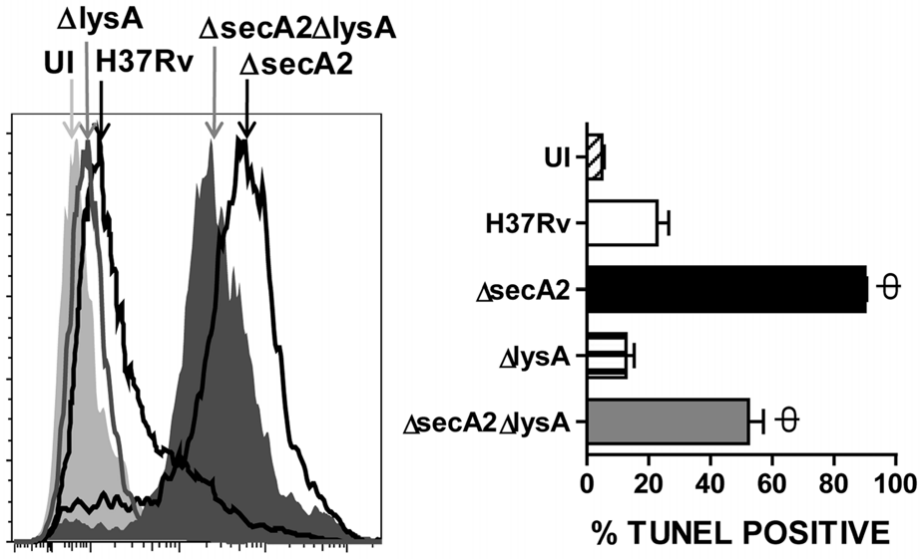
Induction of apoptosis in macrophages by infection with *M. tuberculosis* Δ*secA2*Δ*lysA*. Human THP-1 cells were infected with wild type *M. tuberculosis* (H37Rv) or the *M. tuberculosis* mutant strains Δ*secA2*, Δ*lysA*, or Δ*secA2*Δ*lysA* strains at an MOI of 10. Uninfected (UI) cells were used as a control. Cells were stained after 72 hours infection for TUNEL and analyzed by FACS and representative histograms of TUNEL staining are shown. Quantitation by FACS of cells positive for TUNEL is summarized in the graph. Asterisks indicate statistical significance compared to H37Rv (Ф, p<0.001; one-way ANOVA).

The *secA2* gene encodes an accessory secretion factor required for the export of superoxide dismutase protein (SodA), an enzyme that catalyzes conversion of superoxide anions to hydrogen peroxide. In previous work we have shown that deletion of *secA2* gene results in enhanced apoptosis of infected macrophage, an effect that was reversed by the restoration of secretion of SodA through a *secA2* independent pathway [9]. To test whether the *ΔsecA2ΔlysA* has retained this pro-apoptotic property, transformed human monocyte/macrophage cells (THP-1 cells) were infected with at an MOI of 10 bacilli per macrophage with H37Rv, Δ*secA2*, *ΔlysA*, or *ΔsecA2ΔlysA.* At 72 hours following infection, quantitative assessment of apoptosis by staining for DNA strand breakage (TUNEL) showed that both Δ*secA2* and *ΔsecA2ΔlysA* demonstrated high levels of apoptosis, whereas WT *M. tuberculosis* and the single mutant auxotroph *ΔlysA* did not (Figure 2). These results indicated that the defect in apoptosis inhibition resulting from the loss of *ΔsecA2* was retained in the *ΔsecA2ΔlysA* double mutant.

### Augmented CD8^+^ T cell priming following immunization with *ΔsecA2ΔlysA*

**Figure 3:**
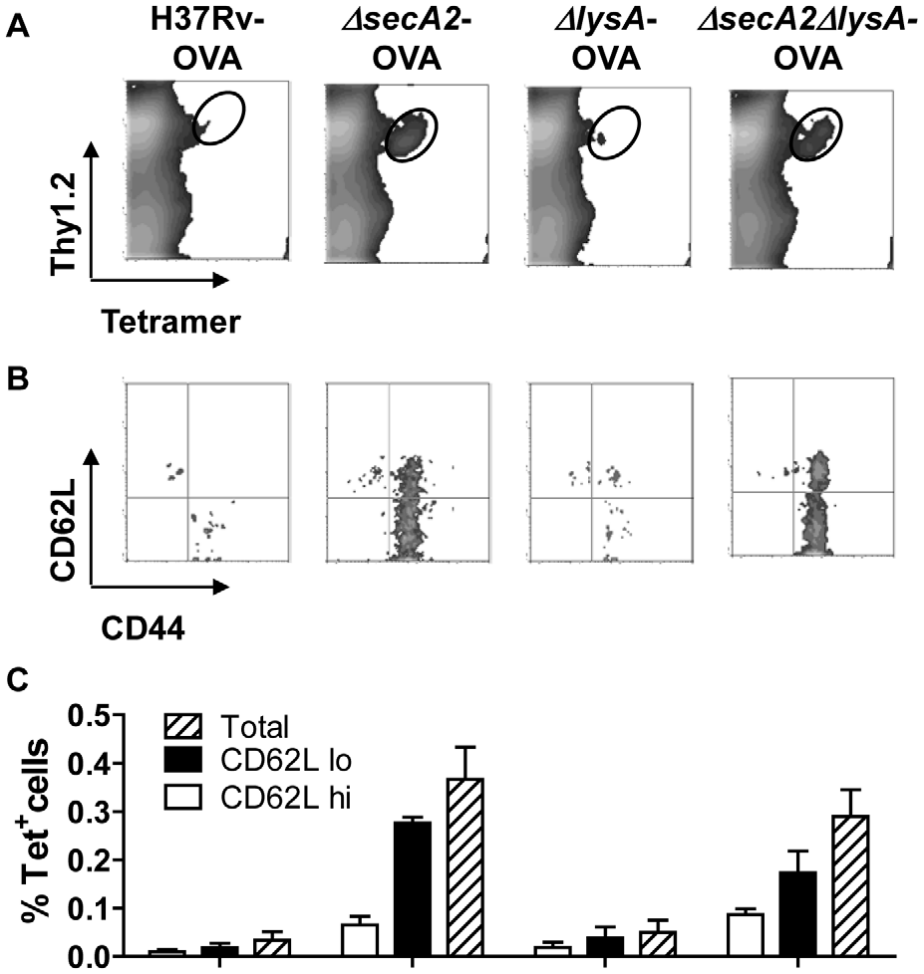
Augmented memory T cell populations following immunization with *M. tuberculosis* Δ*secA2*Δ*lysA*. C57BL/6 mice were injected intravenously with 5×10^5^ OT-I splenocytes 24 hours prior to subcutaneous immunization with either mycobacteria expressing OVA. The animals were sacrificed 5 weeks post immunization, and splenocytes were stained for five color fluorescence analysis by flow cytometry using antibodies specific for Thy1.2, B220, CD62L and CD44 plus SIINFEKL-loaded H-2K^b^ tetramer. (A) FACS analysis of total B220 negative events among 1.5×10^6^ total lymphocytes, showing proportions of Thy1.2^+^ cells staining with tetramer (circled). (B) Dot plots show expression of CD44 and CD62L on cells gated for tetramer staining as in (A) from representative mice infected with the indicated bacterial strains. (C) The graph shows percentages of tetramer positive cells expressing either high levels of CD62L (central memory T cells) or low levels of CD62L (effector memory T cells).

**Figure 4:**
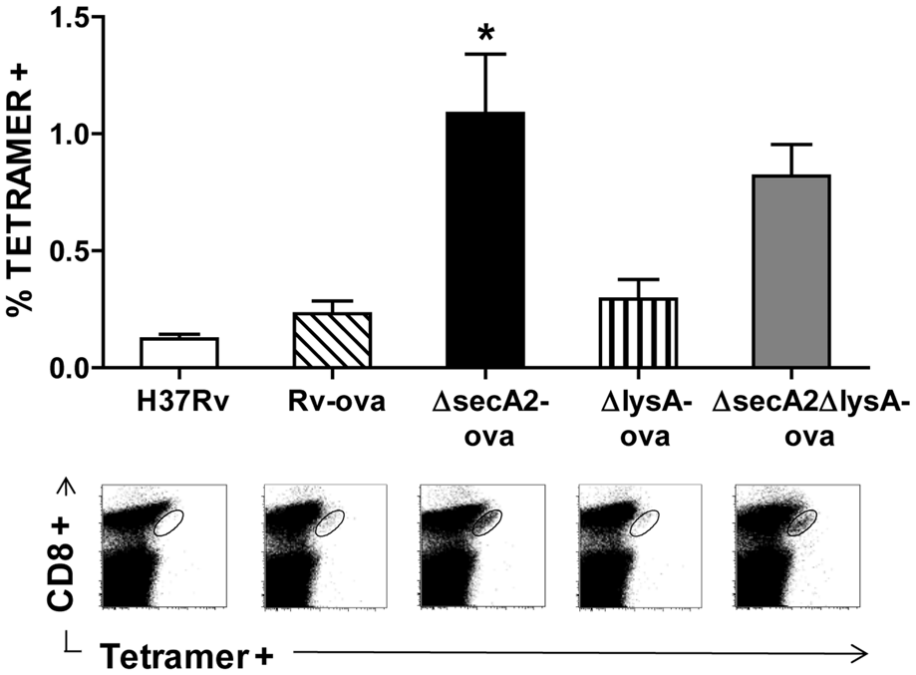
Enhanced priming of endogenous SIINFEKL-specific CD8^**+**^ T cells following immunization with Δ*secA2*Δ*lysA.* The frequencies of SIINFEKL-specific CD8^+^ T cells in spleens from mice infected with the indicated strains of *M. tuberculosis* were determined. The percentage of CD8^+^ cells staining with SIINFEKL-loaded H-2K^b^ tetramers and representative FACS plots are shown for cohorts of four mice that were analyzed 7 days after intravenous infection. Asterisks indicate statistical significance compared to Rv-ova (* <0.05; one-way ANOVA test).

As illustrated in earlier work, an important consequence of blocking apoptosis is an effective reduction of the priming of CD8^+^ T cell response to *M. tuberculosis*. *M. tuberculosis* antigens are produced within a phagosomal compartment which is sequestered from the classical cytoplasmic pathway of antigen processing. As with other vacuolar pathogens, such as *Salmonella*, display of these antigens on MHC class I molecules likely occurs by the uptake and presentation of apoptotic material by bystander dendritic cells (DC) [13]. Consistent with this idea, we have previously shown that mice vaccinated with the Δ*secA2* mutant strain exhibited a marked enhancement of CD8^+^ T cell priming. To test whether the *ΔsecA2ΔlysA* mutant strain retained the enhanced immunogenicity observed in the parental Δ*secA2* strain, we used a CD8^+^ T cell adoptive transfer strategy similar to that described previously [9]. Recipient mice injected with low numbers (5×10^5^) of OT-I T cells were vaccinated subcutaneously with recombinant *M. tuberculosis* strains expressing the 19-kDa lipoprotein-SIINFEKL fusion protein. This experimental design enabled accurate quantification of antigen-specific CD8^+^ T cells by tetramer staining in mice vaccinated with H37Rv-OVA, Δ*secA2*-OVA, *ΔlysA-*OVA, and *ΔsecA2ΔlysA-*OVA. As anticipated, mice vaccinated with H37Rv-OVA and Δ*lysA*-OVA showed little detectable expansion of transferred T cells, while the pro-apoptotic strains, Δ*secA2*-OVA, and *ΔsecA2ΔlysA-*OVA induced significant expansion of antigen specific CD8^+^ T cells (Figure 3A). We also measured expression of CD62 ligand (CD62L) and CD44 on tetramer^+^ cells to distinguish between naive (CD44^low^CD62L^high^), central memory (CD44^high^CD62L^high^), and effector memory cells (CD44^high^CD62L^low^). Our results showed enhanced expansion of both subsets in mutant strains deficient in *secA*2 (Figure 3B, C). In addition, we were able to demonstrate a measurable induction of endogenous CD8^+^ T cells in mice vaccinated intravenously with Δ*secA2*-OVA and *ΔsecA2ΔlysA-*OVA, with little detectible expansion of tetramer positive cells in mice vaccinated with H37Rv-OVA and Δ*lysA*-OVA (Figure 4).

### Protective immunity against virulent *M. tuberculosis* challenge following vaccination with Δ*secA2*Δ*lysA*

**Figure 5:**
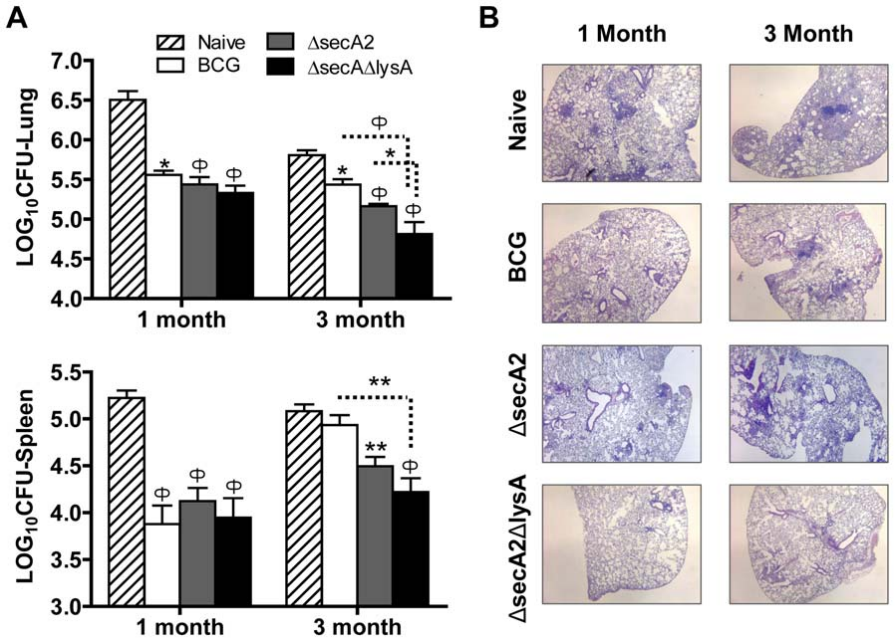
Protective immunity against virulent *M.tuberculosis* challenge in mice following vaccination with *ΔsecA2ΔlysA*. (A) C57BL/6 mice were vaccinated subcutaneously with saline (naïve) or with 1×10^6^ BCG, *M. tuberculosis* Δ*secA2* or Δ*secA2*Δ*lysA*. All mice were challenged by aerosol 2 months later with 50–100 CFU of virulent *M. tuberculosis* (Erdman strain). Graphs show means and standard errors of colony forming units (CFU) of *M. tuberculosis* in lungs and spleens at 1 and 3 months after challenge for groups of five mice. Asterisks indicate statistical significance compared to unvaccinated group or between bracketed groups (*, *p*<0.05; **, *p*<0.01; Ф, *p*<0.001; two-way ANOVA). (B) Lungs of mice vaccinated and challenged with virulent *M. tuberculosis* as in (A) were examined histologically at 1 month and 3 months post-challenge. More severe spreading lung lesions with extensive granulomatous pneumonia and consolidation were observed in unvaccinated mice as compared to mice vaccinated with BCG, Δ*secA2*, or *ΔsecA2ΔlysA* **.**

As discussed above, although Δ*lysA* has a compelling safety profile in immunocompromised animals, and performs much better than BCG in our studies conducted in SCID mice, its poor growth *in vivo* comes at the cost of weaker immunogenicity. In order to determine whether the enhanced T cell priming observed with *ΔsecA2ΔlysA* correlated with improved performance as a live vaccine strain, we conducted immunization and challenge studies designed to evaluate potential vaccine efficacy of the double mutant. C57BL/6 mice that were either naive or immunized 8 weeks earlier by subcutaneous immunization with live Δ*secA2*Δ*lysA*, Δ*secA2* or BCG (Pasteur strain) were challenged by low-dose aerosol infection with virulent *M. tuberculosis* (Erdman). In naive mice, substantial growth in the lungs and dissemination to spleens were detected within 1 month of challenge. In contrast, vaccination with Δ*secA2*Δ*lysA*, Δ*secA2* and BCG considerably reduced *M. tuberculosis* bacillary loads in both lungs and spleens of aerosol-challenged mice as compared with naive controls (Figure 5A).

Immunization with Δ*secA2* and Δ*secA2*Δ*lysA* also showed a more prolonged effect on control of *M. tuberculosis* infection, which was still apparent 3 months after challenge. More importantly, the protection evoked by Δ*secA2*Δ*lysA* immunization was significantly greater than that obtained with BCG vaccination, as measured by bacterial burden in both the lung and spleen at 3 months post challenge (0.66-log reduction in lungs of Δ*secA2*Δ*lysA* versus BCG vaccinated mice; *P*<0.001, 0.68-log reduction in spleens Δ*secA2*Δ*lysA* versus BCG vaccinated mice; *P*<0.01). Interestingly, Δ*secA2*Δ*lysA* vaccinated mice also showed significantly lower lung bacillary loads than mice vaccinated with Δ*secA2* (0.35-log reduction in lungs of Δ*secA2*Δ*lysA* versus Δ*secA2* vaccinated mice; *P*<0.05) (Figure 5A), an effect that may relate to the enhanced stability of memory cells in response to non-persistent live vaccines [14], [15], [16]. The one month time point represents the peak bacillary burden and the 3 month time point represents the set point of the bacillary burden in the chronic phase of infection. Histopathological examination of the lungs from mice immunized with BCG, Δ*secA2,* or Δ*secA2*Δ*lysA* showed relatively mild inflammation with less severe spreading lung lesions, less extensive granulomatous pneumonia, and reduced consolidation compared with naive mice (Figure 5B).

## Discussion

In recent years a number of attenuated strains of *M. tuberculosis*, many constructed through the engineered deleting of genes important for growth in vivo, have been shown to confer protection against challenge with virulent *M. tuberculosis* [17], [18]. The appeal of auxotrophic mutant strains, from the standpoint of vaccine development, relates to their marked growth attenuation *in vivo* and exceptional safety profile in immunocompromised animals. However, a significant drawback of many of these strains is poor immunogenicity in immunocompetent animals, which appears to be a function of reduced growth and decreased antigen load. Consistent with this view, the Δ*lysA* mutant strain, while shown to be profoundly attenuated in SCID mice, required multiple rounds of immunization to achieve levels of protection comparable to BCG in immunocompetent mice [19]. In contrast, we have shown in the current study that a single immunization with the *ΔsecA2ΔlysA* strain conferred markedly enhanced protection from challenge with virulent *M. tuberculosis* when compared to BCG. A similar result was previously observed for the parental *ΔsecA2* strain, which exhibited enhanced CD8^+^ T cell priming when compared with wild-type *M. tuberculosis*, while conferring enhanced protection to vaccinated mice and guinea pigs compared to BCG. Enhanced immunogenicity of *ΔsecA2* correlated with the loss of apoptosis inhibition by the mutant strain. This proapoptotic phenotype was preserved in the Δ*secA2*Δ*lysA* strain, along with the enhanced CD8^+^ T cell stimulating effect. In the present study, we confirmed our previous finding that *ΔsecA2* conferred enhanced protection against virulent challenge when compared with BCG, and demonstrated that vaccination with the Δ*secA2*Δ*lysA* double mutant strain also gave protection that was significantly better than that achieved with BCG. In fact, Δ*secA2*Δ*lysA* vaccination led to a significantly greater reduction in CFU in the lungs of immunocompetent mice at 3 months, when compared with the parental *ΔsecA2* strain.

The explanation for this augmented protective efficacy of Δ*secA2*Δ*lysA* vaccination may relate to the inability this strain to persist *in vivo*, leading to more rapid antigen clearance after T cell priming and more stable memory T cell populations [20]. This is a substantial difference from the parental Δ*secA2* single mutant, which is known to be only moderately attenuated for growth in mice and most likely persists at least at low levels for many weeks and perhaps permanently [21]. Research on T cell responses to viral pathogens including LCMV, Hepatitis C, and SIV, suggests that the persistence of antigen stimulation associated with chronic infection can lead to the functional impairment or ablation of effector and memory CD8^+^ T cell responses. In this model, too little antigen leads to suboptimal responses, whereas an over-abundance or prolonged expression of antigen leads to a suboptimal reservoir of memory T cells [22], [23], [24], [25]. Through the pairing of the augmented CD8+ T cell priming properties of the Δ*secA2* mutation with the restrained growth imparted by Δ*lysA*, we may have achieved a more favorable formulation to generate robust, yet stable, memory responses in vaccinated animals.

A salient advantage of the auxotrophic double mutant, illustrated here, is its marked attenuation in immunocompromised mice. Due to the high incidence of HIV-infection in regions where TB is endemic, safety of immunocompromised animal models has become an important consideration for experimental vaccines. Even BCG, widely regarded as a safe vaccine, has been shown to progress to disease in a significant subpopulation of HIV infected individuals. In a study of BCG vaccinated HIV-positive children conducted in 2003, 10% of mycobacterial disease was attributed to BCG [26]. HIV infection leads to a disproportionate loss of CD4^+^ T cells, which in experimental models have been shown to be essential for controlling infection with BCG [27]. Although CD4^+^ T cells are critical for protective anti-TB protective immunity [28], CD8^+^ T cells have been shown to play an important role in the control of TB, particularly in CD4-deficient mice [5], [6], [29]. Thus, the *ΔsecA2ΔlysA* strain, with an improved safety profile and augmented CD8^+^ T cell immunogenicity, may serve as an effective substitute for BCG in the HIV infected population.

Consistent with results from our previous work characterizing the immunogenicity of Δ*secA2,* the mechanisms underlying the augmented vaccine properties of *ΔsecA2ΔlysA* appear to stem, at least in part, from a loss of apoptosis inhibition, enhanced CD8^+^ T cell priming and memory T cell generation. In addition, by coupling the moderately attenuating Δ*secA2* mutation with Δ*lysA* we were not only able to achieve a superior safety profile, but also significantly improved control of infection relative to the parental strain. The rational design of novel, live attenuated vaccines for tuberculosis depends on integrated disruption of multiple pathogen virulence strategies. This study clearly demonstrates a proof of this principle by combining *ΔsecA2* with a second mutation that interferes with virulence of *M. tuberculosis*, thus generating a novel attenuated candidate mycobacterial vaccine strain with significantly improved safety and efficacy.

## Materials and Methods

### Bacterial strains and growth media

Virulent *M. tuberculosis* strains H37Rv (obtained from Trudeau Institute), Erdman, *M. tuberculosis* Δ*secA2* mutant (mc^2^3112), and *ΔlysA* have been described previously [30]. *M. tuberculosis* strains were grown on Middlebrook 7H9 broth or 7H10 agar containing 10% (vol/vol) oleic acid–albumin-dextrose-catalase (OADC; BD Diagnostics-Diagnostic Systems), 0.5% glycerol, and 0.05% (vol/vol) Tween-80. For *ΔlysA and ΔsecA2lysA,* the culture medium was supplemented with L-lysine *(*80 µg/ml). The *E. coli* strain DH5α was used for cloning purposes and grown on Luria-Bertani agar or broth (Fisher Scientific) at 37°C. Ampicillin (50 µg/ml), hygromycin (50 µg/ml for mycobacterial selection or 150 µg/ml for *E. coli*), and kanamycin (20 µg/ml for mycobacterial selection or 40 µg/ml for *E. coli*) were used to select for recombinant strains.

### Construction of recombinant mycobacterial strains

The double mutant strain Δ*secA2ΔlysA* was constructed using specialized transduction to disrupt the chromosomal copy of the *lysA* gene of an unmarked *secA2* clone, as described [30]. To generate OVA expressing strains of wild type *M. tuberculosis* (H37Rv-OVA) or of mutants (Δ*secA2*-OVA, Δ*lysA*-OVA, and Δ*secA2*Δ*lysA*-OVA), the wild type and mutant strains were transformed with the plasmid p19OVA which encodes the *M. tuberculosis* 19kD lipoprotein fused at the C-terminus with the sequence for ovalbumin that includes the MHC class I presented epitope SIINFEKL [9]. Transformations of *M. tuberculosis* were performed according to a published protocol [31].

### Apoptosis studies in THP-1 cells

THP-1 cells (ATCC) were grown in RPMI-1640 (Gibco BRL; Invitrogen) supplemented with 10% FBS, 1% HEPES, 1% nonessential amino acids and essential amino acids. THP-1 cells were treated with 8 ng/ml PMA (Sigma-Aldrich) in order to induce differentiation into macrophage-like cells. Differentiated THP-1 cells were plated in 6-well plates at 2×10^6^ cells per well and allowed to adhere overnight. Dispersed bacilli were incubated with cells for 3 hours using a multiplicity of infection (MOI) of 10 bacteria to 1 THP-1 cell. The cells were then washed twice with PBS and incubated at 37°C in a 5% CO_2_ incubator for 72 hours. The presence of apoptotic cells in the cell cultures was analyzed with In Situ Cell Death Detection Kit, Fluorescence (Roche Diagnostics), following the manufacturer's instructions.

### T cell activation studies

C57BL/6 mice received 5×10^5^ splenocytes from OVA-specific TCR transgenic OT-1 mice (Taconic/National Institute of Allergy and Infectious Diseases [NIAID]) intravenously, and then infected subcutaneously 24 hours later with 1×10^6^ CFU H37Rv-OVA, Δ*secA2*-OVA, Δ*lysA*-OVA, or Δ*secA2*Δ*lysA*-OVA. At 5 weeks post immunization, splenocytes were isolated and stained with PE-labeled peptide-loaded (SIINFEKL) H-2K^b^ tetramers in combination with antibodies specific to Thy1.2, CD44, CD62L and B220 (all antibodies were purchased from BD Biosciences). Percentages of lymphocytes stained with tetramer and expressing markers consistent with central (CD44^high^CD62L^high^) or effector (CD44^high^CD62L^low^) memory cells were determined by flow cytometry. For detection of endogenous OVA responsive T cells, mice were infected intravenously with 1×10^7^ CFU H37Rv-OVA, Δ*secA2*-OVA, Δ*lysA*-OVA, or Δ*secA2lysA*-OVA. After 7 days splenocytes were isolated and stained with PE-labeled peptide-loaded (SIINFEKL) H-2K^b^ tetramers in combination with antibodies specific to CD8.

### Vaccination and challenge studies

All animal studies were approved by the institutional animal care and use committees of the Albert Einstein College of Medicine (A3312-01), the Center for Biologics Evaluation and Research, and the Duke University (A071-10-03). C57BL/6 mice were vaccinated subcutaneously with 1×10^6^ CFU of *M. tuberculosis* Δ*secA2*, Δ*secA2*Δ*lysA*, or *M. bovis* BCG-Pasteur as described previously [32]. Aerogenic challenge was performed 2 months later using a Glas-Col inhalation chamber to deliver 50–100 CFU per animal of *M. tuberculosis* Erdman strain. Mice were sacrificed at 1 and 3 months after challenge. Lungs and spleens of individual mice were aseptically removed and homogenized separately in 5 ml normal saline plus 0.05% Tween-80 using a Seward Stomacher 80 blender (Tekmar). The homogenates were diluted serially and plated on Middlebrook 7H10 agar. Lung tissues were processed for histopathology using standard paraffin fixation, sectioning, and H&E staining. In the survival study, animals infected with *M. tuberculosis* Erdman were observed daily until they died or became moribund and were euthanized.

### Statistics

Two-way ANOVA with Bonferroni's post test and one-way ANOVA with the Tukey post test were used for analyses using GraphPad Prism version 5 for Windows (GraphPad; [www.graphpad.com]). P values of less than 0.05 were considered significant.
